# Supplementary material for: Genome editing in animals with minimal PAM CRISPR-Cas9 enzymes
Source: Nat Commun. 2022 May 12;13:2601. doi: 10.1038/s41467-022-30228-4 (PMC9098488; doi:10.1038/s41467-022-30228-4)
Supplement: Supplementary file 1 — Supplementary Information [file 41467_2022_30228_MOESM1_ESM.pdf]

Supplementary Information

Genome editing in animals with minimal PAM CRISPR-Cas9 enzymes

Jeremy Vicencio, Carlos Sánchez-Bolaños, Ismael Moreno-Sánchez, David Brena, Charles E. Vejnar, Dmytro Kukhtar, Miguel Ruiz-López, Mariona Cots-Ponjoan, Alejandro Rubio, Natalia Rodrigo Melero,Jesús Crespo-Cuadrado, Carlo Carolis, Antonio J. Pérez-Pulido, Antonio J. Giráldez, Benjamin P. Kleinstiver, Julián Cerón, Miguel A. Moreno-Mateos

Supplementary Figure 1. **Vicencio, Sanchez-Bolaños, et al.**

a

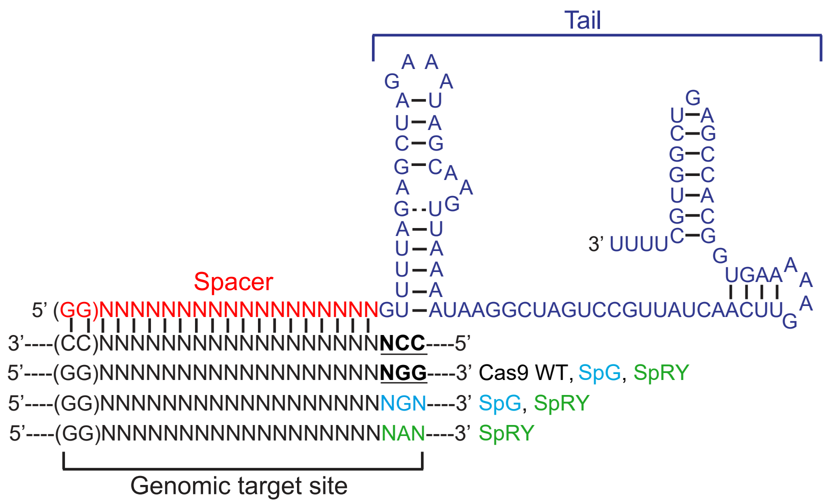

b

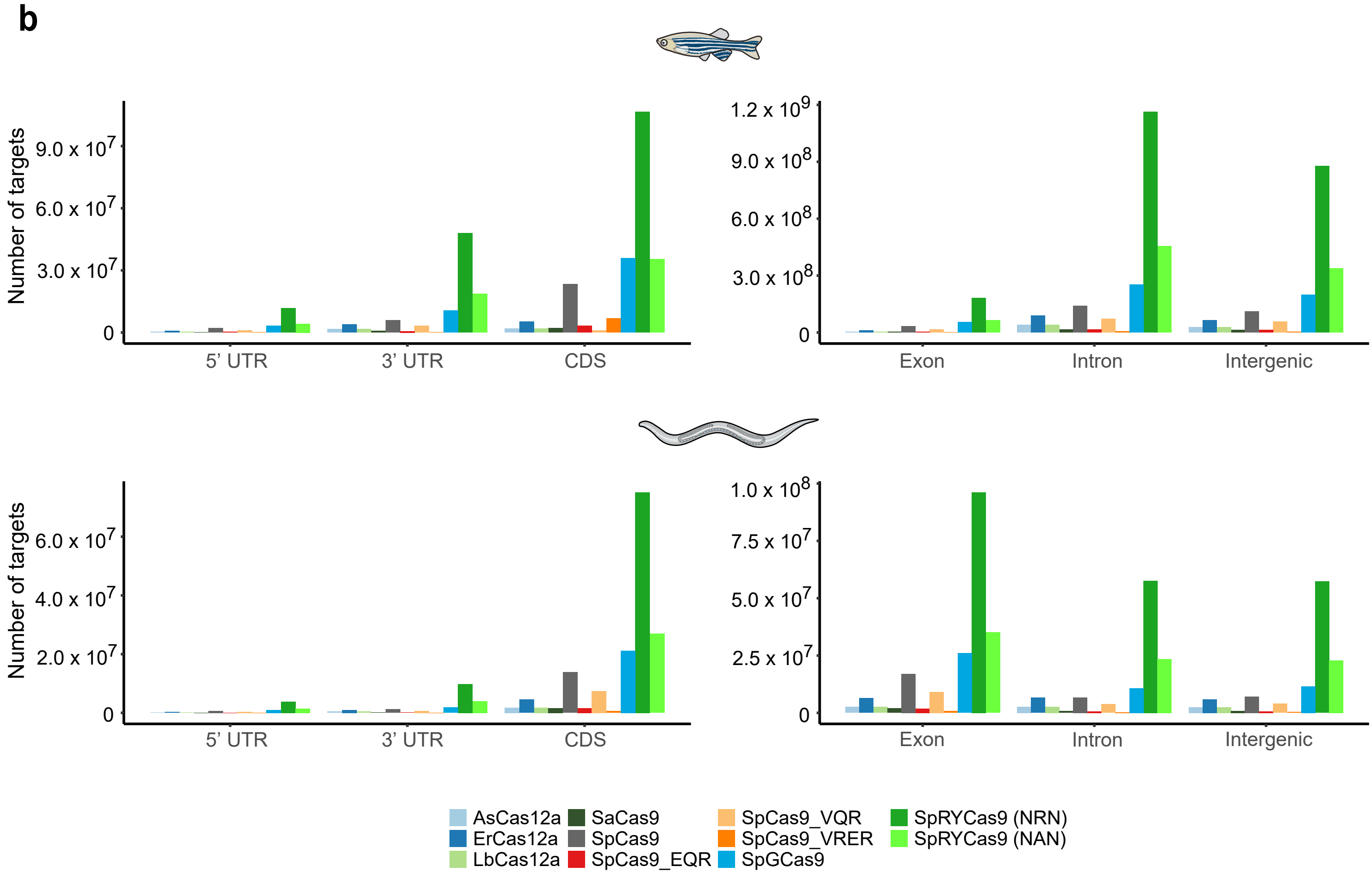

Supplementary Figure 2. Vicencio, Sanchez-Bolaños, et al.

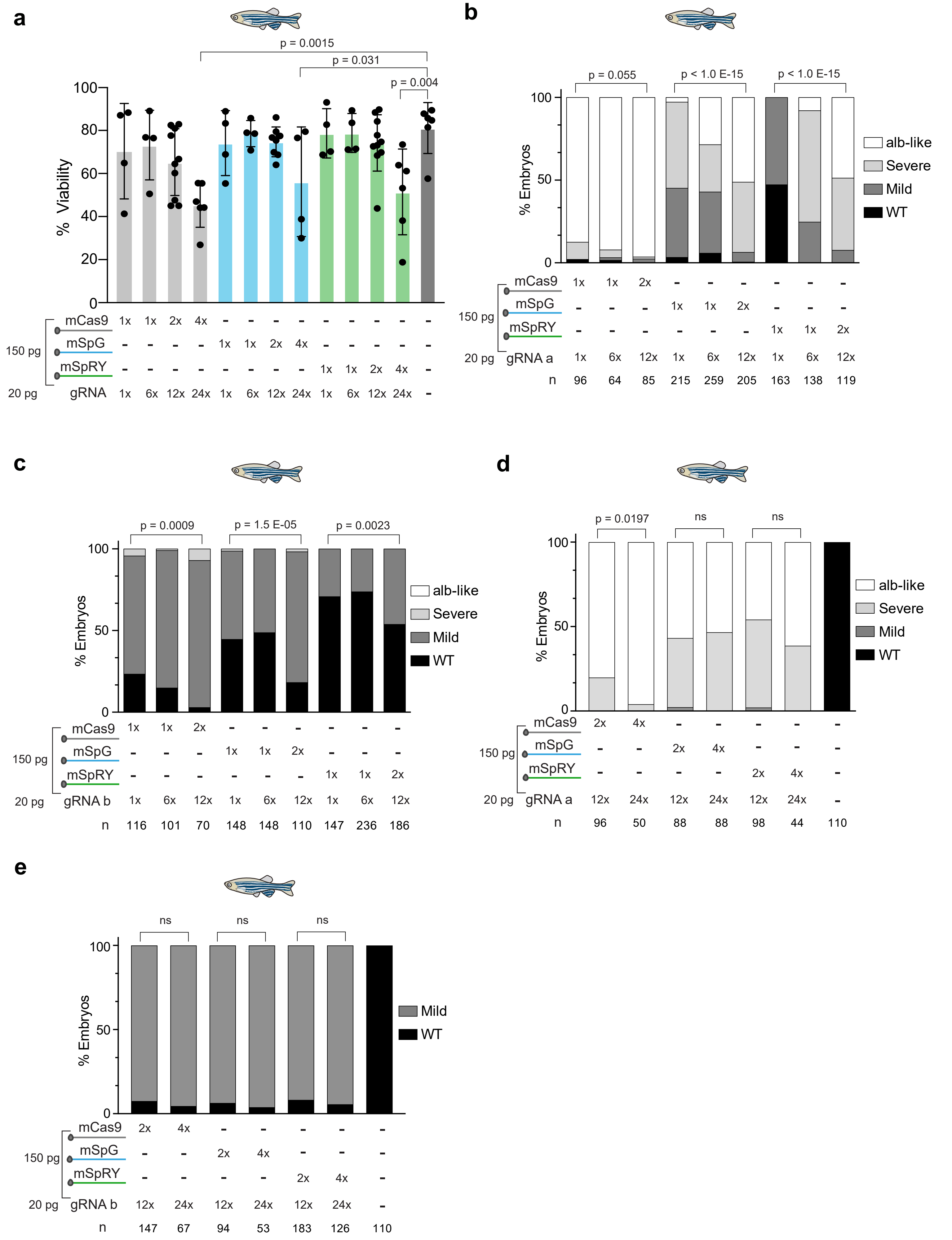

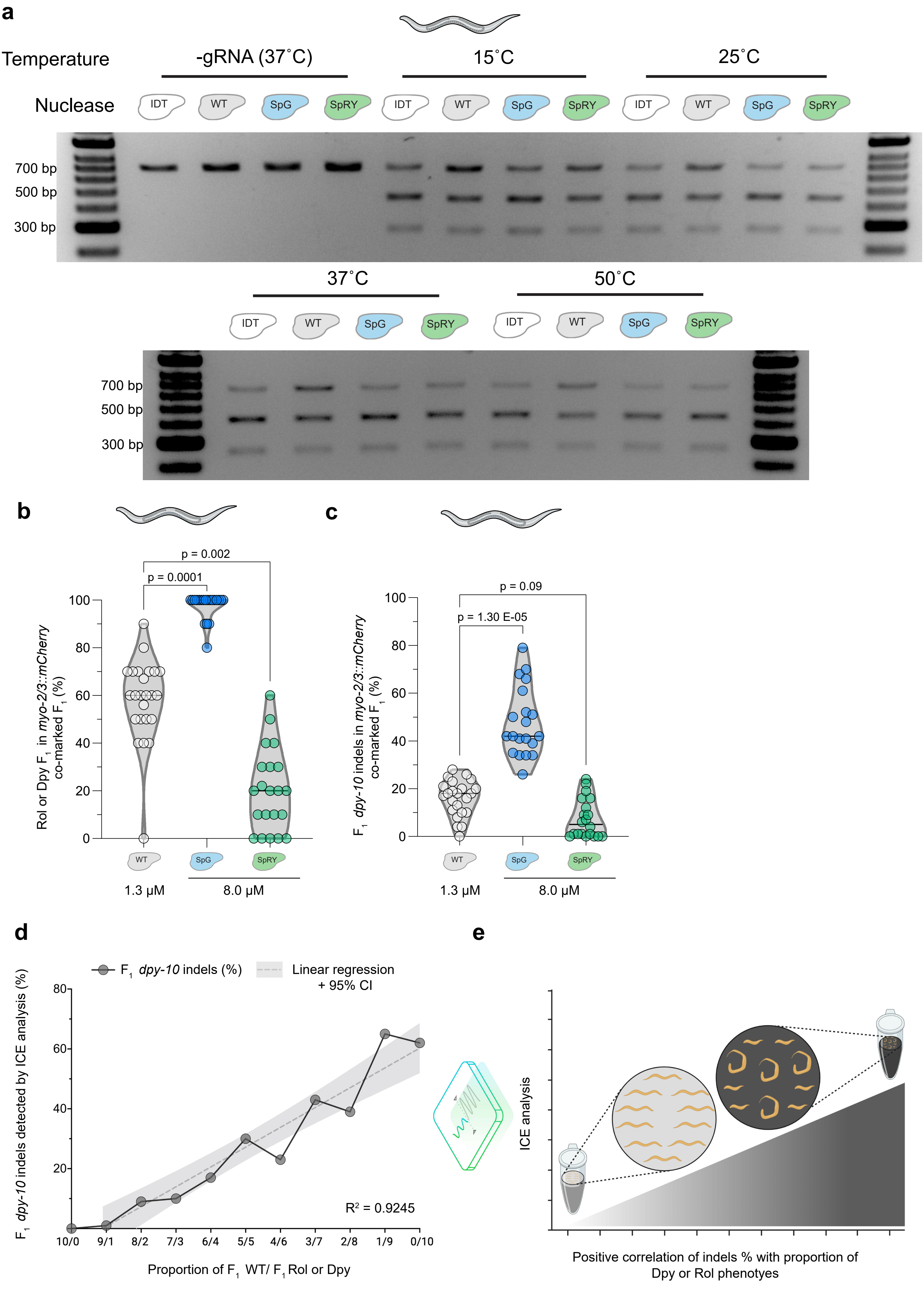
a
Temperature
-gRNA (37˚C)
15˚C
25˚C
Nuclease
IDT
WT
SpG
SpRY
700 bp
500 bp
300 bp
37˚C
50˚C
b
p = 0.002
p = 0.0001
Rol or Dpy F1 in myo-2/3::mCherry co-marked F1 (%)
1.3 μM
8.0 μM
c
p = 0.09
p = 1.30 E-05
F1 dpy-10 indels in myo-2/3::mCherry co-marked F1 (%)
d
F1 dpy-10 indels (%)
Linear regression + 95% CI
F1 dpy-10 indels detected by ICE analysis (%)
R² = 0.9245
10/0
9/1
8/2
7/3
6/4
5/5
4/6
3/7
2/8
1/9
0/10
Proportion of F1 WT/ F1 Rol or Dpy
e
ICE analysis
Positive correlation of indels % with proportion of Dpy or Rol phenotyes

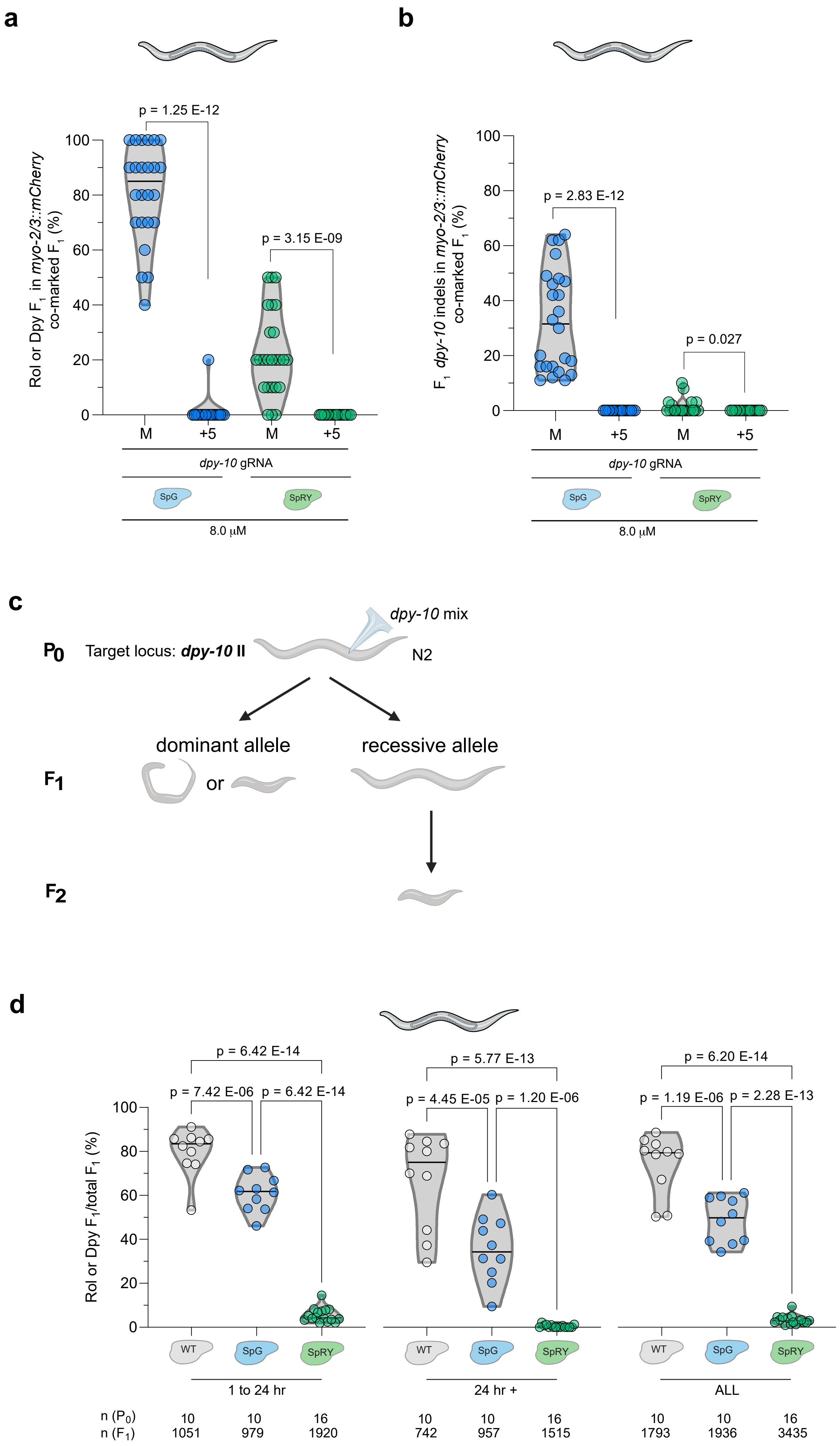
a
p = 1.25 E-12
p = 3.15 E-09
Rol or Dpy F1 in myo-2/3::mCherry co-marked F1 (%)
100
80
60
40
20
0
M
+5
M
+5
dpy-10 gRNA
SpG
SpRY
8.0 µM
b
p = 2.83 E-12
p = 0.027
F1 dpy-10 indels in myo-2/3::mCherry co-marked F1 (%)
c
dpy-10 mix
P0 Target locus: dpy-10 II
N2
dominant allele
recessive allele
F1
or
F2
d
p = 6.42 E-14
p = 7.42 E-06
p = 6.42 E-14
p = 5.77 E-13
p = 4.45 E-05
p = 1.20 E-06
p = 6.20 E-14
p = 1.19 E-06
p = 2.28 E-13
Rol or Dpy F1/total F1 (%)
WT
SpG
SpRY
1 to 24 hr
24 hr +
ALL
n (P0) 10 10 16 10 10 16 10 10 16
n (F1) 1051 979 1920 742 957 1515 1793 1936 3435

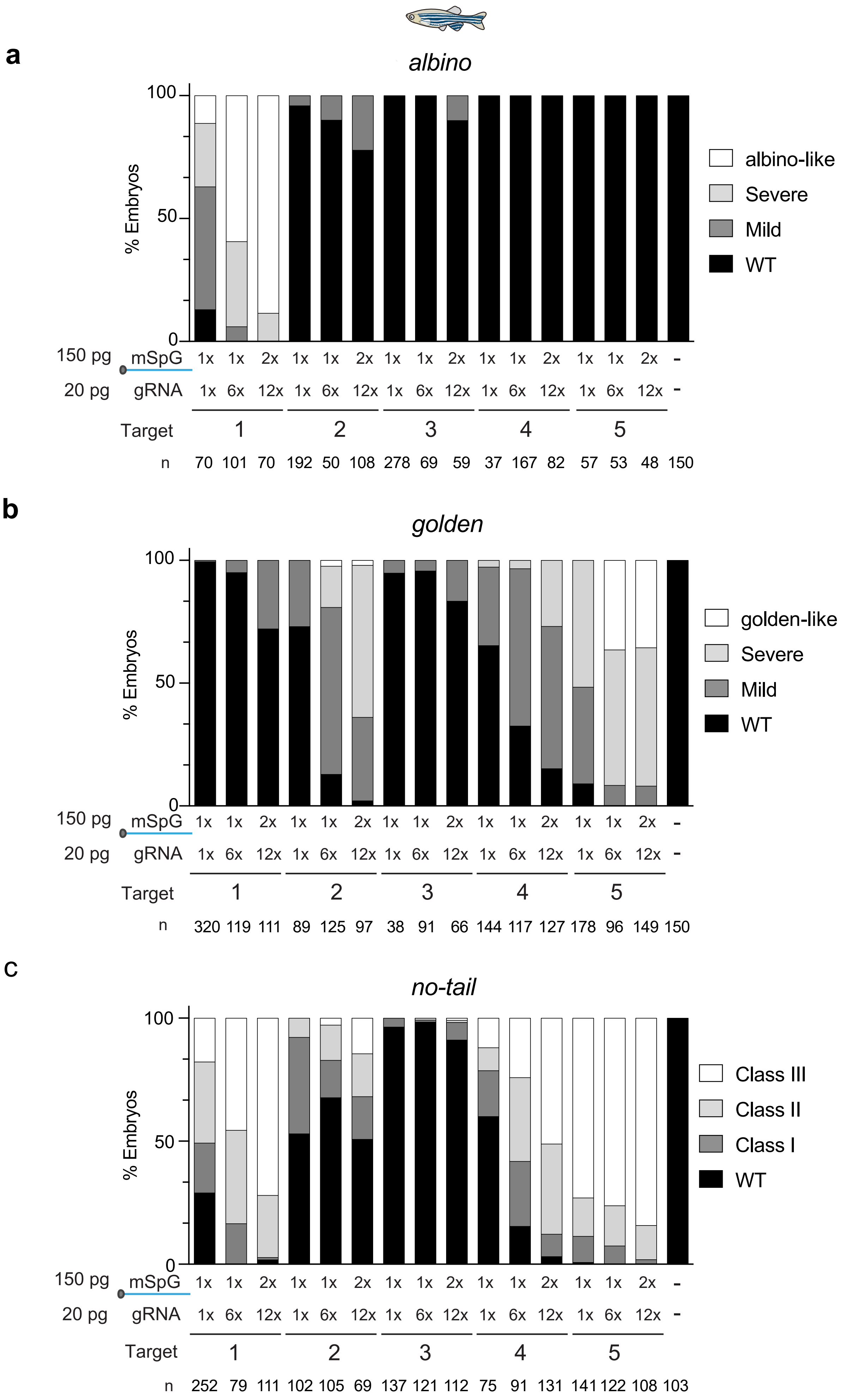
a
albino
100
50
0
% Embryos
albino-like
Severe
Mild
WT
150 pg mSpG 1x 1x 2x 1x 1x 2x 1x 1x 2x 1x 1x 2x 1x 1x 2x -
20 pg gRNA 1x 6x 12x 1x 6x 12x 1x 6x 12x 1x 6x 12x 1x 6x 12x -
Target 1 2 3 4 5
n 70 101 70 192 50 108 278 69 59 37 167 82 57 53 48 150
b
golden
100
50
0
% Embryos
golden-like
Severe
Mild
WT
150 pg mSpG 1x 1x 2x 1x 1x 2x 1x 1x 2x 1x 1x 2x 1x 1x 2x -
20 pg gRNA 1x 6x 12x 1x 6x 12x 1x 6x 12x 1x 6x 12x 1x 6x 12x -
Target 1 2 3 4 5
n 320 119 111 89 125 97 38 91 66 144 117 127 178 96 149 150
c
no-tail
100
50
0
% Embryos
Class III
Class II
Class I
WT
150 pg mSpG 1x 1x 2x 1x 1x 2x 1x 1x 2x 1x 1x 2x 1x 1x 2x -
20 pg gRNA 1x 6x 12x 1x 6x 12x 1x 6x 12x 1x 6x 12x 1x 6x 12x -
Target 1 2 3 4 5
n 252 79 111 102 105 69 137 121 112 75 91 131 141 122 108 103

Supplementary Figure 6. **Vicencio, Sanchez-Bolaños, et al.**

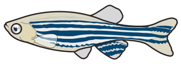

a

Target *golden* 5

```
WT  TGGGTTTCAGGTCTTGGTCTCTCGCAGGATGTTGCTGGAGCCACGTTTATGGCTGCGGGGAGTTCGGCTCCAGAG
D3  TGGGTTTCAGGTCTTGGTCTCTCGC---ATGTTGCTGGAGCCACGTTTATGGCTGCGGGGAGTTCGGCTCCAGAG
D4  TGGGTTTCAGGTCTTGGTCTCTCG----ATGTTGCTGGAGCCACGTTTATGGCTGCGGGGAGTTCGGCTCCAGAG
D7  TGGGTTTCAGGTCTTGGTCTCTCG-------TTGCTGGAGCCACGTTTATGGCTGCGGGGAGTTCGGCTCCAGAG
D7  TGGGTTTCAGGTCTTGGTCTCTCGC-------TGCTGGAGCCACGTTTATGGCTGCGGGGAGTTCGGCTCCAGAG
I1  TGGGTTTCAGGTCTTGGTCTCTCGCNAGGATGTTGCTGGAGCCACGTTTATGGCTGCGGGGAGTTCGGCTCCAGA
```

Target *albino* 1

```
WT   CTACTGTAGGTCGTCATGGGGCCGAAGGAGACCGTACATACTCTTACTGGGGATTCTGATGTTAGTGGGCATGAC
D10  CTACTGTAGGTCGTCATGGGGCCGA----------ACATACTCTTACTGGGGATTCTGATGTTAGTGGGCATGAC
D10  CTACTGTAGGTCGTCATGGGGCC----------GTACATACTCTTACTGGGGATTCTGATGTTAGTGGGCATGAC
D9   CTACTGTAGGTCGTCATGGGGCCGA---------TACATACTCTTACTGGGGATTCTGATGTTAGTGGGCATGAC
D9   CTACTGTAGGTCGTCATGG---------AGACCGTACATACTCTTACTGGGGATTCTGATGTTAGTGGGCATGAC
D18  CTACTGTAGGTCGTCATGGGG------------------ACTCTTACTGGGGATTCTGATGTTAGTGGGCATGAC
D9   CTACTGTAGGTCGTCATGGGG---------ACCGTACATACTCTTACTGGGGATTCTGATGTTAGTGGGCATGAC
D9   CTACTGTAGGTCGTCATGGGGCCG---------GTACATACTCTTACTGGGGATTCTGATGTTAGTGGGCATGAC
D4   CTACTGTAGGTCGTCATGGGG----AGGAGACCGTACATACTCTTACTGGGGATTCTGATGTTAGTGGGCATGAC
I1   CTACTGTAGGTCGTCATGGGGCCGANAGGAGACCGTACATACTCTTACTGGGGATTCTGATGTTAGTGGGCATGA
```

Target *no-tail* 1

```
WT   ACGGGTGCTTTCATCCAGTGCGCGCCGAAGTTGGGTGAGTCCGGGTGGATGTAGACGCAGCTCGGGCTTTGGGGT
D3   ACGGGTGCTTTCATCCAGTGCGCG---AAGTTGGGTGAGTCCGGGTGGATGTAGACGCAGCTCGGGCTTTGGGGT
D3   ACGGGTGCTTTCATCCAGTGCGCGC---AGTTGGGTGAGTCCGGGTGGATGTAGACGCAGCTCGGGCTTTGGGGT
D5   ACGGGTGCTTTCATCCAGTGCGC-----AGTTGGGTGAGTCCGGGTGGATGTAGACGCAGCTCGGGCTTTGGGGT
D5   ACGGGTGCTTTCATCCAGTGCGCG-----GTTGGGTGAGTCCGGGTGGATGTAGACGCAGCTCGGGCTTTGGGGT
D1   ACGGGTGCTTTCATCCAGTGCGCG-CGAAGTTGGGTGAGTCCGGGTGGATGTAGACGCAGCTCGGGCTTTGGGGT
D12  ACGGGTGCTTTCATC------------AAGTTGGGTGAGTCCGGGTGGATGTAGACGCAGCTCGGGCTTTGGGGT
I1   ACGGGTGCTTTCATCCAGTGCGCGCNCGAAGTTGGGTGAGTCCGGGTGGATGTAGACGCAGCTCGGGCTTTGGGG
```

b

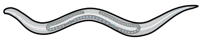

Target *dpy-10*

```
WT     CTTCAATACGGCAAGATGAGAATGACTGGAAACCGTACCGCTCGTGGTGCCTATGGTAGCGGAGCTTCACATGGCTTCAG
D9     CTTCAATACGGCAAGATGAGAATGACTGGAAAC---------CGTGGTGCCTATGGTAGCGGAGCTTCACATGGCTTCAG  SpGe R
D3     CTTCAATACGGCAAGATGAGAATGACTGGAAACCGTA---CTCGTGGTGCCTATGGTAGCGGAGCTTCACATGGCTTCAG  SpGe R
D5     CTTCAATACGGCAAGATGAGAATGACTGGAAAC-----CGCTCGTGGTGCCTATGGTAGCGGAGCTTCACATGGCTTCAG  SpGe r
D5-I1  CTTCAATACGGCAAGATGAGAATGACTGGAAACT----CGCTCGTGGTGCCTATGGTAGCGGAGCTTCACATGGCTTCAG  SpGe r
D9     CTTCAATACGGCAAGATGAGAATGACTGGAAA---------TCGTGGTGCCTATGGTAGCGGAGCTTCACATGGCTTCAG  SpGe r
```

Target *wrmScarlet*-NAC2

```
WT     GGACCACTCCCATTCTCCTGGGACATCCTCTCCCCACAATTCATGTACGGATCCCGTGCCTTCACCAAGCACCCAGCCGA
D8     GGACCACTCCCATTCTCCTGGGAC--------CCCACAATTCATGTACGGATCCCGTGCCTTCACCAAGCACCCAGCCGA  WT Cas9
D9     GGACCACTCCCATTCTCCTGGGAC----------CACAATTCATGTACGGATCCCGTGCCTTCACCAAGCACCCAGCCGA  SpRY
D3     GGACCACTCCCATTCTCCTGGGACATCCTCT---CACAATTCATGTACGGATCCCGTGCCTTCACCAAGCACCCAGCCGA  SpRY
D5     GGACCACTCCCATTCTCCTGGGACAT-----CCCCACAATTCATGTACGGATCCCGTGCCTTCACCAAGCACCCAGCCGA  SpRY
D4-I3  GGACCACTCCCATTCTCCTGGGACATCCTC----ACAAATTCATGTACGGATCCCGTGCCTTCACCAAGCACCCAGCCGA  SpRY
```

Target *wrmScarlet*-NAC3

```
WT     ACAAGCAATCCTTCCCAGAGGGATTCAAGTGGGAGCGTGTCATGAACTTCGAGGACGGAGGAGCCGTCACCGTCACCCAAG
D11    ACAAGCAATCCTTCCCAGAGGGATTCAAGTGGGAG-----------CTTCGAGGACGGAGGAGCCGTCACCGTCACCCAAG  SpRY
```

Target *wrmScarlet*-NGH1

```
WT      GACCAGTCATGCAAAAGAAGACCATGGGATGGGAGGCCTCCACCGAGCGTCTCTACCCAGAGGACGGAGTCCTCAAGGGAG
D9      GACCAGTCATGCAAAAGAAGACCATGGGATGGGAG---------GAGCGTCTCTACCCAGAGGACGGAGTCCTCAAGGGAG  SpG
D10-I1  GACCAGTCATGCAAAAGAAGACCATGGGATGGGAGG----------GGCGTCTCTACCCAGAGGACGGAGTCCTCAAGGGAG  SpG
D3      GACCAGTCATGCAAAAGAAGACCATGGGATGGGAGGCCT---CCGAGCGTCTCTACCCAGAGGACGGAGTCCTCAAGGGAG  SpRY
D6      GACCAGTCATGCAAAAGAAGACCATGGGATGGGAGG------CCGAGCGTCTCTACCCAGAGGACGGAGTCCTCAAGGGAG  SpRY
```

Supplementary Figure 7.

Vicencio, Sanchez-Bolaños, et al.

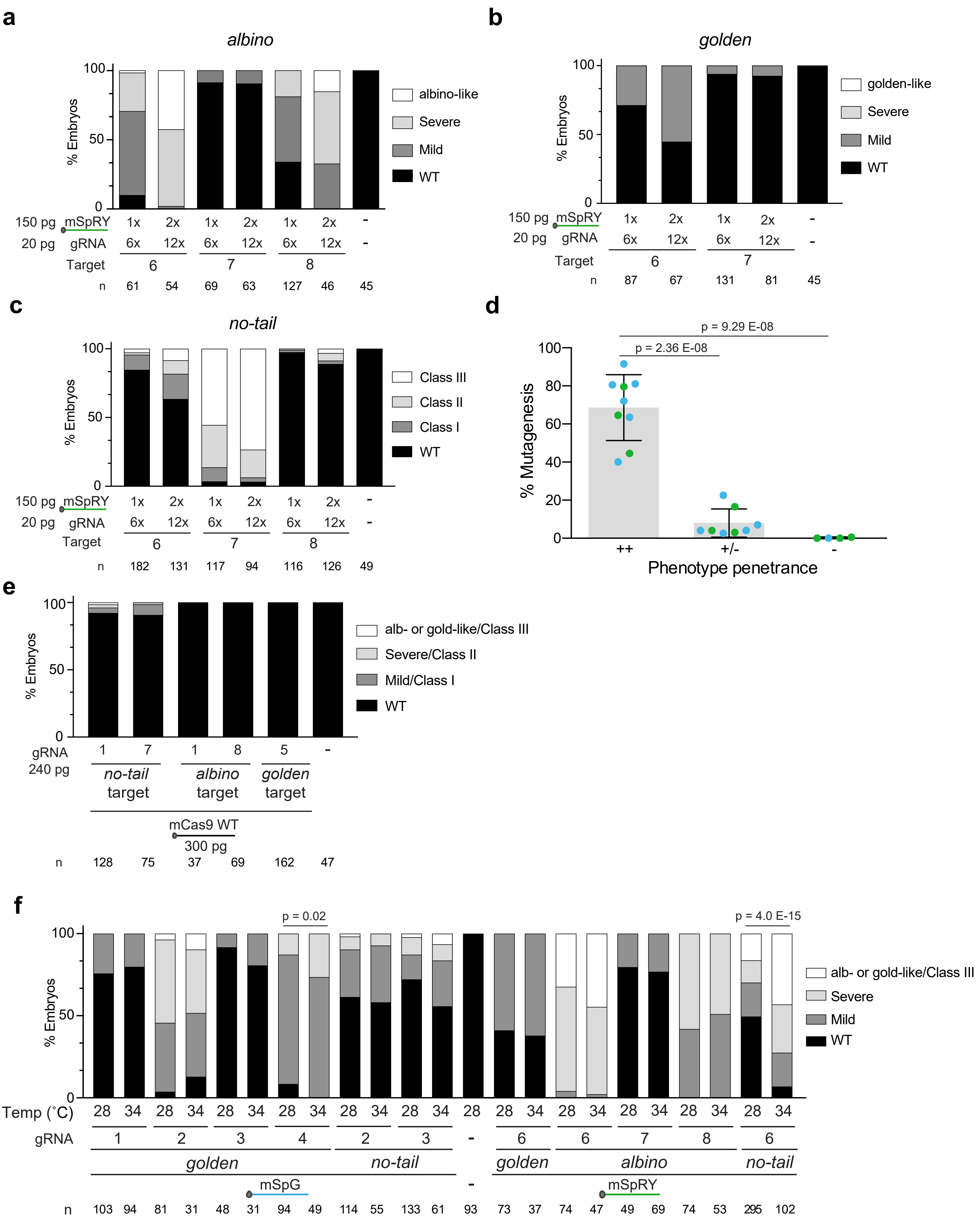

Supplementary Figure 8.

Vicencio, Sanchez-Bolaños, et al.

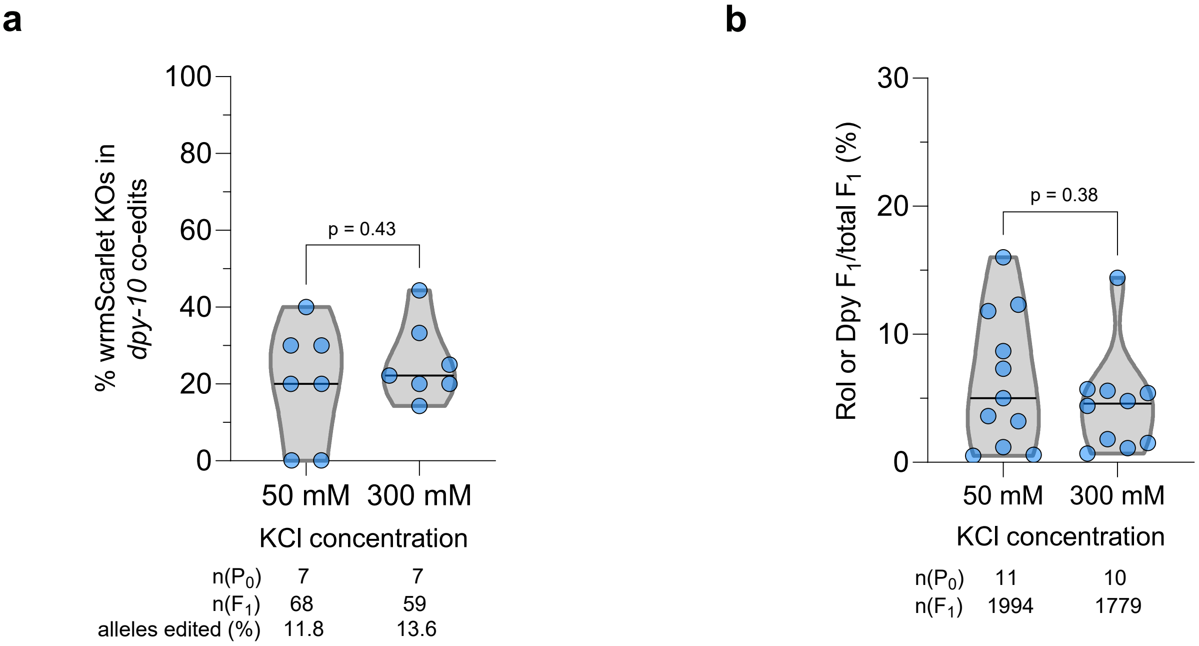

Supplementary Figure 9.

Vicencio, Sanchez-Bolaños, et al.

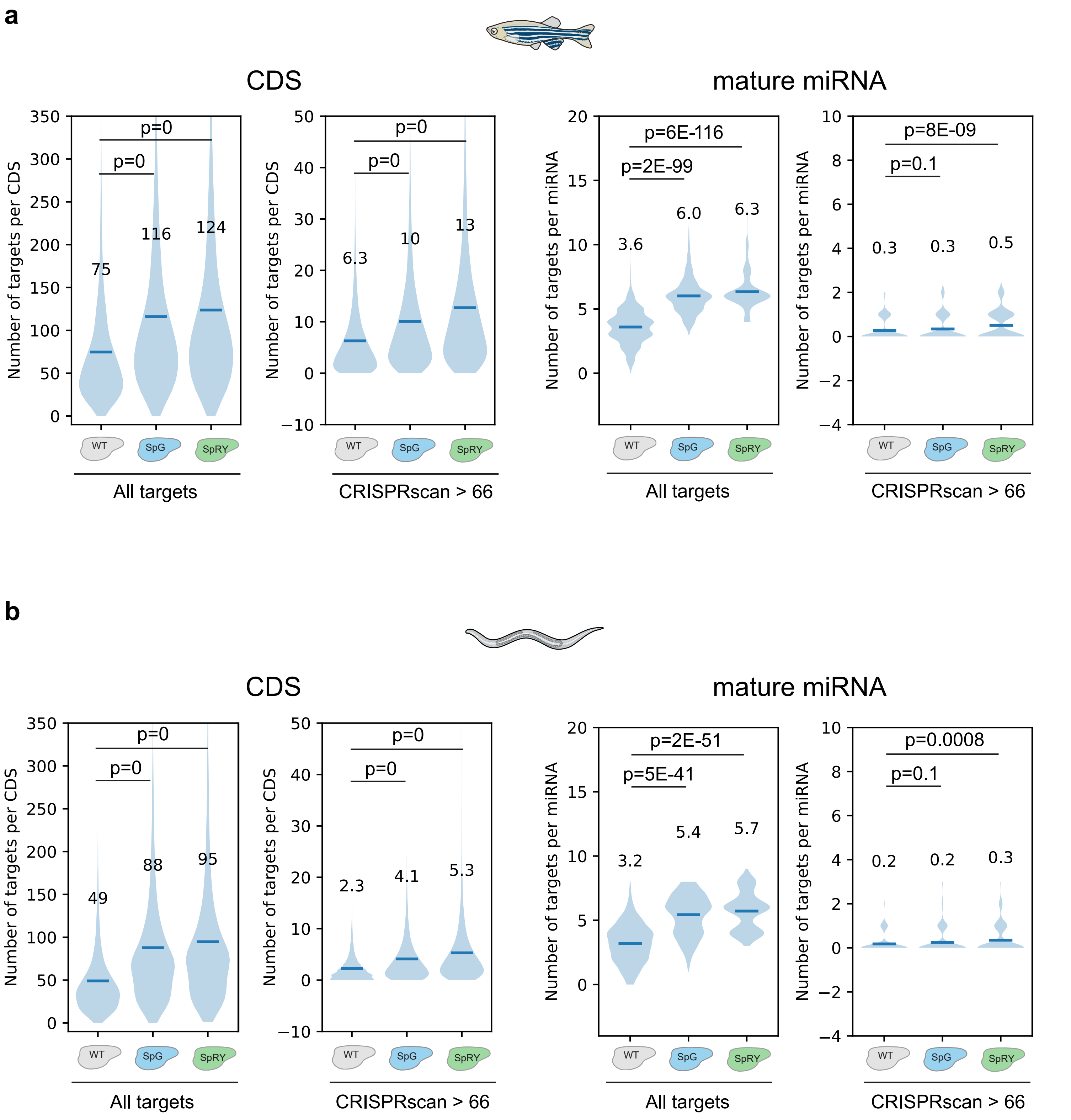

Supplementary Figure 10. **Vicencio, Sanchez-Bolaños, et al.**

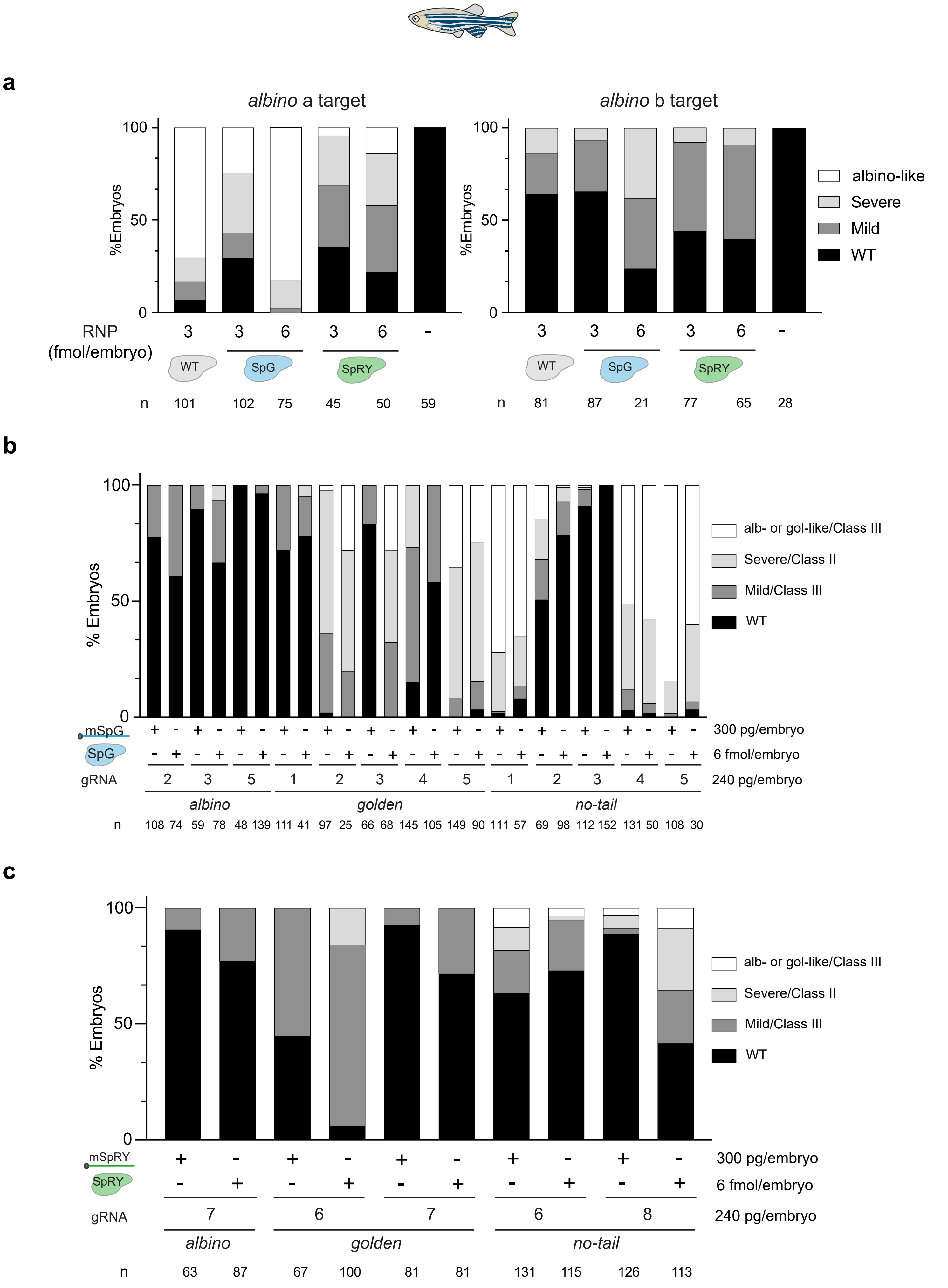

Supplementary Figure legends

Supplementary Figure 1. Features of CRISPR-Cas9 systems

a. Scheme showing a gRNA (spacer in red, tail in blue) binding to the genomic target site (black) with the PAM sequence 5'-NGG (black, bold, and underlined), 5'-NGN (blue) and 5'-NAN (green) downstream for Cas9 WT-SpG-SpRY, SpG-SpRY or SpRY only, respectively. *In vitro* transcribed gRNAs were used for this study where a 5' GG requirement in the gRNA (between parenthesis) was needed for efficient transcription [56]. Adapted from Moreno-Mateos et al., 2017 [20].

b. Bar graphs showing number of targets of the indicated CRISPR endonucleases in the zebrafish and *C. elegans* genomes, separated in 5' UTRs, 3' UTRs, CDS, exons, introns, and intergenic regions. AsCas12a: *Acidaminococcus* spp. Cas12a; ErCas12a: *Eubacterium rectale* Cas12a; LbCas12a: *Lachnospiraceae bacterium* Cas12a; SaCas9: *Staphylococcus aureus* Cas9; SpCas9: *Streptococcus pyogenes* Cas9; SpCas9 EQR: *S. pyogenes* Cas9 EQR mutant; SpCas9VQR: *S. pyogenes* Cas9 VQR mutant; SpCas9VRER: *S. pyogenes* Cas9 VRER mutant; SpGCas9: *S. pyogenes* Cas9 SpG variant; and SpRYCas9: *S. pyogenes* Cas9 SpRY variant.

Supplementary Figure 2. SpG and SpRY are active nucleases in zebrafish

a. Bar graphs showing the percentage of viability in the injected and non-injected embryos at 24 hpf. The embryos were injected with a mix containing 150 (1x), 300 (2x) or 600 pg (4x) of mRNA coding for Cas9 WT (mCas9, grey), SpG (mSpG, blue), or SpRY (mSpRY, green) and 20 (1x), 120 (6x), 240 (12x) or 480 (24x) pg of gRNA a or b targeting *albino* (**Fig. 1**). Results are shown as averages ± standard error of the mean from at least two independent experiments with each gRNA (a or b) (p-value using one-way ANOVA followed by Dunnett test for comparisons with non-injected control). Each dot represents an independent experiment, $n \geq 4$.

b-e. Phenotypic evaluation of injected embryos with different amounts of mRNAs coding for Cas9 WT (mCas9), SpG (mSpG), or SpRY (mSpRY) and gRNA a (**b,d**) or b (**c,e**) targeting *slc45a2* (*albino*) showing different levels of mosaicism compared to the WT. Data of panels **b** and **c** are from **Fig. 1c,d**. Stacked barplots show the percentage of alb-like (white), severe (light grey), mild (dark grey), and phenotypically WT (black) embryos 48 hpf after injection. The total number of injected embryos (n) is shown below. The results were obtained from at least two independent experiments. The $\chi 2$-test p-value per comparison is shown, ns: no significant difference, $p \geq 0.05$).

Supplementary Figure 3. RNP activity of SpCas9, SpG, and SpRY *in vitro* and *in vivo* in *C. elegans.*

a. IDT Cas9, WT SpCas9, SpG, or SpRY were tested *in vitro* at different temperatures by incubating the RNPs with a *dpy-10* PCR product. Negative controls without gRNA (-gRNA) were tested at 37 °C. Upper bands show uncleaved PCR product at 698 bp. Lower bands show cleaved products. This experiment was performed once.

b-c. Phenotypic (**b**) and mutagenic (**c**) quantification of edits with *dpy-10* matched gRNA and (SpCas9) WT, SpG, and SpRY [n (P0) 24, 20, and 21, respectively]. Mutagenic analysis was performed using ICE [62]. Note that due to the dominance of *dpy-10* alleles, the percentage of indels in the F_1 (c) is lower than Dpy or Rol

phenotypes (b). *myo-2p*::mCherry and *myo-3p*::mCherry were used as co-injection markers. 10 F_1 worms expressing mCherry were singled out and the appearance of Dpy or Rol progeny was screened in the F_2. Each dot represents the editing efficiency in each I P_0 that produced at least ten mCherry-expressing F_1s. Violin plots are represented with individual data points and the median. The results were obtained from a single experiment, with all conditions carried out in parallel injections (Kruskal-Wallis test followed by Dunn's test for multiple comparison p-values).

d. The graph depicts the positive correlation of different proportions of F_1 WT animals and F_1 Rol or Dpy animals in pools of 10 analyzed for the percentage of indels using ICE analysis [62]. Dark gray dots connected with a black line represent the percentage of indels detected by ICE at each known proportion of Dpy or Rol animals in pools of 10. Note that the percentage of indels does not reach 100% because the screened animals were mostly heterozygous F_1s. The gray dotted line shows the linear regression and the light gray background represents the 95% confidence interval (CI) calculated using the Pearson correlation test. R^2 is the Pearson correlation coefficient.

e. Schematic representation of the assay showing the positive correlation of the percentage of indels detected by ICE analysis [62] with known proportions of Dpy or Rol phenotypes. WT and Dpy or Rol F_1 animals are shown in pools of 10.

Supplementary Figure 4. Quantification of *in vivo* specificity of Cas9 variants

a-b. The *in vivo* specificity of SpG and SpRY were tested in *C. elegans* by injecting either a *dpy-10* matched (M) or +5 mismatched gRNA. The graphs show the phenotypic (**a**) and mutagenic (**b**) quantification of the *dpy-10* edits comparing SpG and SpRY under optimized concentrations. Mutagenic analysis was performed using ICE [62]. The fluorescent markers *myo-2p*::mCherry and *myo-3p*::mCherry were used as co-injection markers. F_1 worms expressing mCherry in the pharynx or body wall muscle were singled out and the appearance of Dpy or Rol progeny was screened in the F_2. Each dot represents the editing efficiency (number of F1 worms that segregate Dpy or Rol progeny divided by the number of separated F1s) in each P_0 that produced at least ten mCherry-expressing F_1s. The results were obtained from a single experiment, with all conditions carried out in parallel injections. n (P0) 24, 19, 25 and 19 for SpG(M), SpG(+5), SpRY(M) and SpRY(+5) respectively (Mann-Whitney test p-value).

c. Schematic representation of *in vivo* experiments in *C. elegans* targeting *dpy-10*. Mutations leading to dominant Rol or Dpy phenotypes are detected in the F_1. In some experiments, wild type-like F_1 progeny are singled out to screen for recessive Rol or Dpy phenotypes in the F_2.

d. Comparison of WT Cas9, SpG, and SpRY editing efficiencies in *C. elegans* using the canonical NGG *dpy-10* co-CRISPR gRNA [18] at a nuclease concentration of 1.6 μM. The left panel includes F_1 progeny laid between 1 and 24 hr post-injection. The middle panel includes F_1 progeny laid after 24 hr post-injection. The right panel includes all F_1 progeny, all from the same set of injected P_0s. Each dot represents the editing efficiency in each P_0 that produced at least 100 F_1. The numbers indicate the total number of viable injected P_0s and screened F_1s per group. The results are obtained from two independent

experiments, with all three conditions carried out in parallel injections (One-way ANOVA followed by Tukey's test for multiple comparisons p-values).

Supplementary Figure 5. SpG is an active nuclease at minimal PAM targets in zebrafish.

a-b. Phenotypic evaluation of injected embryos with different amounts of mRNAs coding SpG (mSpG) and gRNAs (1-5) targeting sites with NGH PAMs for *slc45a2* (*albino*) (**a**) or *slc24a5* (*golden*) (**b**) genes. Stacked barplots show the percentage of albino/golden-like (white), severe (light grey), mild (dark grey), and phenotypically WT (black) embryos 48 hpf after injection. The total number of injected embryos (n) is shown below. Data in optimal conditions (300 pg mRNA and 240 pg gRNA per embryo) from **Fig. 2b**. The results were obtained from at least two independent experiments.

c. Phenotypic evaluation of injected embryos with different amounts of mRNAs coding for SpG (mSpG) and gRNAs (1-5) targeting sites with NGH PAMs for *tbxta* (*no-tail*) gene. Stacked barplots show the percentage of class III (white), class II (light grey), class I (dark grey), and phenotypically WT (black) embryos 28 hpf after injection. The total number of injected embryos (n) is shown below. Data in optimal conditions (300 pg mRNA and 240 pg gRNA per embryo) from **Fig. 2b**. The results were obtained from at least two independent experiments.

Supplementary Figure 6. Examples of mutations obtained with SpG complexes targeting *slc45a2 (albino)*, *slc24a5 (golden)* and *tbxta (no-tail)* genes in zebrafish; and endogenous SpG (SpGe) targeting *dpy-10* and WT Cas9, SpG, and SpRY targeting wrmScarlet in *C. elegans*.

a. Five 24 hpf zebrafish embryos per condition were collected for DNA extraction (see Methods). Different targeting (*golden* 5, *albino* 1, and *no-tail* 1) regions were PCR amplified and sequenced. The reference sequence (WT) is provided as the first sequence in each group of alignments.; The PAM is underlined; D: deletion (-). I: insertion. N: undetermined insertion.

b. Each sequence is derived from individual worms consisting of either F_2 dumpies or F_2 wrmScarlet knockouts. WT, SpG, and SpRY indicate the nuclease used to edit the genome. SpGe indicates that the animals were edited using the endogenously expressed SpG from the germline. For the *dpy-10* alignments, R indicates that the allele is dominant, and r indicates recessive. The reference sequence (WT) is provided as the first sequence in each group of alignments. The PAM is underlined; -: deletion. D: deletion (-). I: insertion.

Supplementary Figure 7. SpRY, but not WT Cas9, is a competent nuclease at minimal PAM targets in zebrafish and phenotype quantification correlates with mutagenesis levels using near PAM-less Cas in zebrafish

a-c. mRNAs coding for SpRY and gRNAs (6-8) targeting NAN PAM sites for *slc45a2* (*albino*) (**a**), *slc24a5* (*golden*) (**b**) or *tbxta* (*no-tail*) (**c**) genes. Stacked barplots show the percentage of phenotypes classes in embryos 28 (no-tail) or 48 hpf (albino and golden) after injection. The results were obtained from at least two independent experiments. Data in optimal conditions (300 pg mRNA and 240 pg gRNA per embryo) from **Fig. 2f**. (n) total number of injected embryos.

d. The level of mutagenesis correlates with the phenotype penetrance. Bar graphs showing percentage of mutagenesis when using SpG (blue dots) and SpRY (green dots) targeting their specific sites with NGH and NAN PAMs.

Results are shown as averages ± standard error of the mean from at least 4 targets ($n \geq 4$). **++** represents highly efficient targets**:** at least 50% of the embryos presented severe (Class II or alb/gol severe mosaic) or extremely severe (Class III or alb/gol like) phenotypes). **+/-** represents low-medium efficient targets: at least 10% of embryos are mosaic. **–** represents no active targets. Data analyzed using one-way ANOVA followed by Tukey's test for multiple comparisons.

e. Phenotypic evaluation of embryos injected with mCas9 and gRNAs that show high activity with mSpG and mSpRY against NGH and NAN PAMs. Stacked barplots show the percentage of phenotypes in embryos 28 and 48 hpf after injection. The results were obtained from at least two independent experiments.

f. Phenotypic evaluation of embryos injected with 300 pg of SpG or SpRY and 240 pg of gRNAs targeting three genes at different sites. Embryos were incubated either at 28 ^{o}C or at 34 ^{o}C for 24 h and then maintained at 28 ^{o}C. Stacked barplots show the percentage of phenotypes in embryos 28 (no tail) and 48 hpf (albino and golden) after injection. The results were obtained from at least two independent experiments. The $\chi 2$-test p-value per comparison is shown only for those with a significant difference ($p < 0.05$).

Supplementary Figure 8. Salt concentration does not affect the editing efficiency of SpG RNP *in vivo*

a. Salt concentration does not affect the editing efficiency of an anti-wrmScarlet:SpG RNP (NGH PAM). RNPs consisting of SpG and an anti-wrmScarlet gRNA at the basal 1.3 µM concentration were injected in the CER541 strain to target the wrmScarlet protein with an NGH PAM. Two injection mixes with a final KCl concentration of either 50 mM or 300 mM were compared. The editing efficiency is defined as the number of F_1 worms exhibiting loss of fluorescence in the F_2 divided by the total number of separated *dpy-10* co-edited F_1s. Each dot represents the editing efficiency in each individual P_0 that produced at least eight Dpy or Rol F_1s. The numbers indicate the total number of viable injected P_0s and screened F_1s per group. The horizontal black lines represent the median of seven P_0s for each salt concentration, and the grey outlines are violin plots. The results were obtained from a single experiment, with both conditions carried out in parallel injections (Student's *t*-test p-value).

b. Salt concentration does not affect the editing efficiency of a *dpy-10*:SpG RNP (NGG PAM). RNPs consisting of SpG and a *dpy-10* gRNA at the basal 1.3 µM concentration were injected in the N2 strain to target the *dpy-10* gene with an NGG PAM. Two injection mixes with a final KCl concentration of either 50 mM or 300 mM were compared. The editing efficiency is defined as the number of F_1 progeny with Rol or Dpy phenotypes divided by the total number of F_1 progeny laid by each P_0. Each dot represents the editing efficiency in each individual P_0 that produced at least 100 F_1. The horizontal black lines represent the median of ten to 11 P_0s for each salt concentration, and the grey outlines are violin plots. The results were obtained from a single experiment, with both conditions carried out in parallel injections (Student's *t*-test p-value).

Supplementary Figure 9. CRISPRscan can help to predict SpG and SpRY highly efficient targets.

Violin plots (kernel density estimation) representing the distribution of the number of total or CRISPRscan score >66 targets found within zebrafish (**a**) and *C. elegans* (**b**) protein coding-sequences (CDS) and mature miRNA genomic sites

for SpCas9 (with NGG PAM), SpG (with NGN PAM) and SpRY (with N[A/G]N PAM). Means of each distribution is shown with horizontal blue line with mean value written on top. Comparisons are tested using Mann Whitney U tests.

Supplementary Figure 10. RNP can increase SpG/SpRY activity in zebrafish.

a. Phenotypic evaluation of injected embryos with 3 or 6 µM (fmol/embryo) of RNP complexes containing Cas9 (WT, grey), SpG (blue), or SpRY (green) proteins and gRNA a and b for *slc45a2* (*albino*) gene. 6 fmol of RNP is equivalent to 1 ng of protein and 240 pg of gRNA per embryo. Stacked barplots show the percentage of albino-like (white), severe (light grey), mild (dark grey), and phenotypically WT (black) embryos 48 hpf after injection. The total number of injected embryos (n) is shown below. The results were obtained from at least two independent experiments.

b-c. Comparison of the activity between mSpG and SpG RNP (**b**) or mSpRY and SpRY RNP (**c**) using different gRNAs targeting genomic sites with NGH or NAN PAMs respectively, in three genes (*slc45a2*, *albino*; *slc24a5*, *golden*; *tbxta*, *no-tail*). Phenotypic evaluation of embryos injected with 300 pg of mSpG/mSpRY and 240 pg of each gRNA or embryos injected with 6 µM (6 fmol/embryo) of RNP complexes containing SpG/SpRY protein and gRNA. mSpG and mSpRY data from **Supplementary Fig. 5 and 7, respectively**. Stacked barplots show the percentage of albino-like/golden-like/class III (white), severe/class II (light grey), mild/class I (dark grey), and phenotypically wild-type (WT; black) embryos 28 and 48 h post fertilization (hpf) after injection. The total number of injected embryos (n) is shown below. The results were obtained from at least two independent experiments.
