# Supplementary material for: Genome editing in animals with minimal PAM CRISPR-Cas9 enzymes
Source: Nat Commun. 2022 May 12;13:2601. doi: 10.1038/s41467-022-30228-4 (PMC9098488; doi:10.1038/s41467-022-30228-4)
Supplement: Supplementary file 2 — Reporting Summary [file 41467_2022_30228_MOESM2_ESM.pdf]

Corresponding author(s): Julián Cerón and Miguel A. Moreno-Mateos

Last updated by author(s): Apr 12, 2022

Reporting Summary

Nature Portfolio wishes to improve the reproducibility of the work that we publish. This form provides structure for consistency and transparency in reporting. For further information on Nature Portfolio policies, see our Editorial Policies and the Editorial Policy Checklist.

Statistics

For all statistical analyses, confirm that the following items are present in the figure legend, table legend, main text, or Methods section.

n/a	Confirmed	
☐	☒	The exact sample size (*n*) for each experimental group/condition, given as a discrete number and unit of measurement
☐	☒	A statement on whether measurements were taken from distinct samples or whether the same sample was measured repeatedly
☐	☒	The statistical test(s) used AND whether they are one- or two-sided<br>*Only common tests should be described solely by name; describe more complex techniques in the Methods section.*
☒	☐	A description of all covariates tested
☐	☒	A description of any assumptions or corrections, such as tests of normality and adjustment for multiple comparisons
☐	☒	A full description of the statistical parameters including central tendency (e.g. means) or other basic estimates (e.g. regression coefficient) AND variation (e.g. standard deviation) or associated estimates of uncertainty (e.g. confidence intervals)
☐	☒	For null hypothesis testing, the test statistic (e.g. *F*, *t*, *r*) with confidence intervals, effect sizes, degrees of freedom and *P* value noted<br>*Give P values as exact values whenever suitable.*
☒	☐	For Bayesian analysis, information on the choice of priors and Markov chain Monte Carlo settings
☒	☐	For hierarchical and complex designs, identification of the appropriate level for tests and full reporting of outcomes
☒	☐	Estimates of effect sizes (e.g. Cohen's *d*, Pearson's *r*), indicating how they were calculated

Our web collection on statistics for biologists contains articles on many of the points above.

Software and code

Policy information about availability of computer code

Data collection	No software was used for data collection.
Data analysis	SSPS 25.0 (IBM, Armonk, NY, USA), Prism (GraphPad Software v9, La Jolla, CA, USA), or R programming language.

For manuscripts utilizing custom algorithms or software that are central to the research but not yet described in published literature, software must be made available to editors and reviewers. We strongly encourage code deposition in a community repository (e.g. GitHub). See the Nature Portfolio guidelines for submitting code & software for further information.

Data

Policy information about availability of data

All manuscripts must include a data availability statement. This statement should provide the following information, where applicable:

- Accession codes, unique identifiers, or web links for publicly available datasets
- A description of any restrictions on data availability
- For clinical datasets or third party data, please ensure that the statement adheres to our policy

Genome sequences from C. elegans (v. WBcel235) and D. rerio (v. GRCz11) were downloaded from Ensembl database. Source data are provided with this paper. Key plasmids have been deposited in Addgene. Other relevant data are available within the article and its supplementary Information files or from corresponding authors upon reasonable request.

Field-specific reporting

Please select the one below that is the best fit for your research. If you are not sure, read the appropriate sections before making your selection.

☒ Life sciences ☐ Behavioural & social sciences ☐ Ecological, evolutionary & environmental sciences

For a reference copy of the document with all sections, see nature.com/documents/nr-reporting-summary-flat.pdf

Life sciences study design

All studies must disclose on these points even when the disclosure is negative.

Sample size	For C. elegans experiments, sample sizes were not predetermined based on statistical methods, but were chosen according to the standards when microinjecting adult hermaphrodites (at least 20 individuals to produce enough F1 for statistical analyses). No statistical methods were used to predetermine sample size for zebrafish experiments.
Data exclusions	We did not exclude any data from consideration.
Replication	Reported results were produced for replicates generating similar results. Zebrafish phenotype or viability data in different injections conditions come from, at least, two independent injection experiments per figure panel. Zebrafish mutagenesis data per specific SpG and SpRY targets are coming from the average of two biological replicates. C. elegans data come from one or two independent experiments per figure panel with all data derived from parallel injections unless otherwise specified in the figure legend.
Randomization	For C. elegans microinjections, individuals from plates were randomly picked. Other kind of randomization was not necessary in this study.
Blinding	Blinding was not necessary in our quantitative experiments.

Reporting for specific materials, systems and methods

We require information from authors about some types of materials, experimental systems and methods used in many studies. Here, indicate whether each material, system or method listed is relevant to your study. If you are not sure if a list item applies to your research, read the appropriate section before selecting a response.

Materials & experimental systems

n/a	Involved in the study	
☒	☐	Antibodies
☒	☐	Eukaryotic cell lines
☒	☐	Palaeontology and archaeology
☐	☒	Animals and other organisms
☒	☐	Human research participants
☒	☐	Clinical data
☒	☐	Dual use research of concern

Methods

n/a	Involved in the study	
☒	☐	ChIP-seq
☒	☐	Flow cytometry
☒	☐	MRI-based neuroimaging

Animals and other organisms

Policy information about studies involving animals; ARRIVE guidelines recommended for reporting animal research

Laboratory animals	Wild-type zebrafish embryos were obtained through natural mating of AB/Tübingen AB/Tu zebrafish of mixed ages (5–18 months). Selection of mating pairs was random from a pool of 20 males and 20 females. All experiments involving zebrafish conform to national and European Community standards for the use of animals in experimentation and were approved by the ethical committees from the University Pablo de Olavide, CSIC, and the Andalusian Government. Zebrafish wild-type strains AB/Tübingen (AB/Tu) were maintained and bred under standard conditions.<br>C. elegans wild type strain Bristol N2 was obtained from the Caenorhabditis Genetics Center (CGC). The strain CER541 gtbp-1(cer149 [gtbp-1::wrmScarlet]) IV was used for wrmScarlet knockout experiments. EG9615 (oxSi1091[mex-5p::Cas9(smu-2 introns) unc-119(+)] II; unc-119(ed3) III), a strain carrying a transgene that expresses SpCas9 in the germline was a gift from Dr. Matthew Schwartz and Dr. Erik Jorgensen.
Wild animals	This study did not involve wild animals
Field-collected samples	This study did not use samples collected from the field.
Ethics oversight	No approval was necessary because invertebrates and zebrafish embryos are not considered as animals for committees regulating ethic in biomedical research.

Note that full information on the approval of the study protocol must also be provided in the manuscript.
