# Supplementary material for: Genome editing in animals with minimal PAM CRISPR-Cas9 enzymes
Source: Nat Commun. 2022 May 12;13:2601. doi: 10.1038/s41467-022-30228-4 (PMC9098488; doi:10.1038/s41467-022-30228-4)
Supplement: Supplementary file 4 — Description of Additional Supplementary Files [file 41467_2022_30228_MOESM4_ESM.pdf]

Title: Supplementary Data1:
Description: PAMs, primers, CRISPR-Cas targets and crRNAs/gRNAs used in this study

Title: Supplementary Data 2:
Description: Detailed information of Figure 4c.
